# Exogenous Melatonin and Abscisic Acid Expedite the Flavonoids Biosynthesis in Grape Berry of *Vitis vinifera* cv. Kyoho

**DOI:** 10.3390/molecules25010012

**Published:** 2019-12-18

**Authors:** Mingyi Yang, Lei Wang, Tarun Belwal, Xiaocheng Zhang, Hongyan Lu, Cunkun Chen, Li Li

**Affiliations:** 1 Key Laboratory for Agro-Products Postharvest Handling of Ministry of Agriculture, Zhejiang Key Laboratory for Agro-Food Processing, College of Biosystems Engineering and Food Science, Zhejiang University, Hangzhou 310058, China; ymy008@zju.edu.cn (M.Y.); wangley@zju.edu.cn (L.W.); tarungbpihed@gmail.com (T.B.); fyzxc@163.com (X.Z.); luhongyan@zju.edu.cn (H.L.); 2 National Engineering Technology Research Center for Preservation of Agricultural Products, Key Laboratory of Postharvest Physiology and Storage of Agricultural Products, Ministry of Agriculture of China, Tianjin 300112, China; cck0318@126.com; 3 Ningbo Research Institute, Zhejiang University, Ningbo 315100, China; 4 National-Local Joint Engineering Laboratory of Intelligent Food Technology and Equipment, Zhejiang Key Laboratory for Agro-Food Processing, Zhejiang Engineering Laboratory of Food Technology and Equipment, Zhejiang University, Hangzhou 310058, China

**Keywords:** grape, melatonin, abscisic acid, flavonoid, *Vitis vinifera*

## Abstract

Grape polyphenols contributing to more than half of the global polyphenol market were well studied; however, how melatonin (MLT), a potential plant hormone, and abscisic acid (ABA) affects polyphenols profile is still poorly understood. To explore whether these hormones are involved in polyphenolic biosynthesis, grape (*Vitis vinifera* cv. Kyoho) was exposed to MLT, ABA, and NDGA (nordihydroguaiaretic acid, an ABA biosynthesis inhibitor) treatments, and 16 polyphenols were identified from grape extracts by high performance liquid chromatography quadrupole time of flight mass spectrometry (HPLC-Q-TOF-MS). Both exogenous MLT and ABA significantly enhanced the biosynthesis of each flavonol and flavanol component, especially catechin, which was almost increased double by 200 µM of MLT treatment. Furthermore, the expression of genes involved in flavonoid biosynthesis, including 4-coumaroyl-CoA synthase, chalcone synthase, flavonoid 3′-hydroxylase, anthocyanin 3′-methyltransferase, flavonol synthase, flavonoid-3-*O*-glucosyltransferase, and flavonoid 3′,5′-methyltransferase were highly up-regulated as well but were down-regulated by NDGA. The present study provided new insights for improving flavonoids accumulation in agricultural production and its underlying mechanism.

## 1. Introduction

Grapevine (*Vitis vinifera* L.) is a rich source of natural antioxidant compounds, mainly polyphenols, which are composed of flavonols, flavanols, and anthocyanins and contributed to more than half of the global polyphenol market [1]. Grape polyphenols are important secondary metabolites with various health-promoting effects, including anti-inflammatory, anti-cancer, anti-irradiation, anti-bacterial, preventing cardiocerebrovascular diseases, and so on [2]. Besides, *Vitis vinifera* L cv. Kyoho is one of the most popular cultivars due to its sweetness, juiciness, and large size, and Kyoho cultivar also contribute significantly to the world fresh table grapes [3].

Since first discovered in the Japanese morning glory, melatonin (MLT) has been widely studied in plants and plays an important role in stress resistance and antioxidation [4]. Exogenous MLT treatment delayed fruit senescence and improved postharvest commercial value, like inhibiting fruit softening, weight loss, decay rates, and respiration rate of various fruit. Also, promoted endogenous MLT biosynthesis and antioxidant system were observed in pear [5], strawberry [6], peach [7], banana [8], fruit, etc. As a functional component in wine, MLT also had synergistic health effects with polyphenols and increased the vasodilation and antioxidation activities [9]. In addition, 50 µM MLT could significantly increase the lycopene level of tomatoes by 5.8 times [10] and maintain the concentrations of total phenolics, flavonoids, and anthocyanins in litchi fruit, contributing to improved antioxidant capacity [11]. In grape berries, it was reported that pre-harvest exogenous MLT treatment significantly increased the polyphenolic content, antioxidant capacity, and related gene expressions, and improved the fruit maturity [12].

Abscisic acid (ABA) is one of the crucial plant hormones, which play vital roles in fruit ripening and development. Studies reported that exogenous ABA promoted fruit coloration, including anthocyanin and flavanol accumulation during fruit ripening in apple [13], citrus [14], grape [15], litchi [16], strawberry [17], and tomato [18]. It was also reported that the transcriptional levels of phenylalanine ammonia-lyase (*PAL*), cinnamate-4-hydroxylase (*C4H*), chalcone synthase (*CHS*), chalcone isomerase (*CHI*), dihydroflavonol 4-reductase (*DFR*), leucoanthocyanidin dioxygenase (*LDOX*) genes involved in polyphenolic biosynthesis were significantly increased by exogenous ABA treatment [16]. Hu et al. observed that 25 mg L^−1^ exogenous ABA promoted flavonoid biosynthetic gene expression and maintained the color of litchi pericarp [16]. The nordihydroguaiaretic acid (NDGA), an ABA biosynthesis inhibitor, was evident to be involved in the regulation of anthocyanin biosynthesis [19]. Moreover, the effect of ABA on transcript expression of genes involved in anthocyanin and flavonoid biosynthesis has been confirmed in strawberry [20].

However, the effect of postharvest exogenous MLT, ABA, and NDGA on the performance of polyphenolic biosynthesis in grapevine is unclear. The present study was to elucidate the polyphenol profiles and try to develop its relationship with gene expression in *Vitis vinifera* cv. Kyoho, in order to provide new insights for the improvement of polyphenol accumulation.

## 2. Results

### 2.1. Grape Morphology and Berry TSS, TA Concentrations

**Figure 1:**
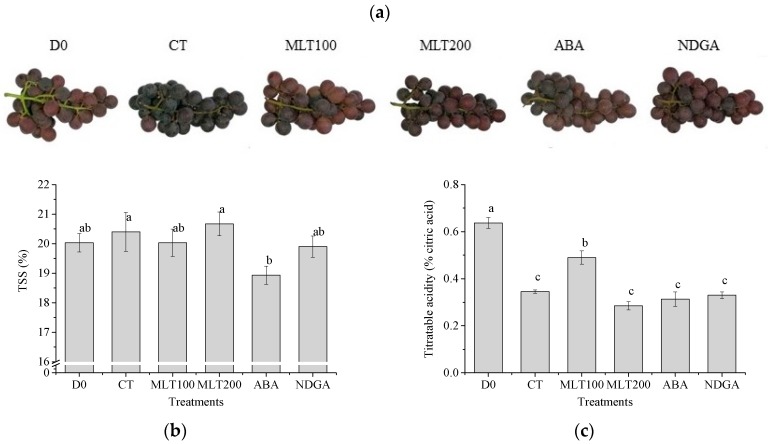
Grape morphology (**a**), Total soluble solid (TSS) content (**b**), and Total acid content (TA) (**c**) of *Vitis vinifera* cv. ‘Kyoho’. Error bars represent the standard deviations of three replicates. Different letters (a–c) on the bars represent significant differences between treatments (*p* < 0.05).

In order to investigate the effects of MLT and ABA on grape acceptance, the morphology of grape bunches at harvest and after storage is shown in Figure 1a. The results showed no obvious difference in the treated grape bunches after three day (d) storage at room temperature. Total soluble solid (TSS), which is a measure for sucrose concentration was found to be non-affected with all treatments, except ABA (Figure 1b), but the total acid (TA) concentration was halved to approximately 0.3% citric acid equivalents after three d storage (Figure 1c). It was interesting to mention that exogenous MLT at a lower concentrations significantly increased the TA concentration by about 0.15% (*p* < 0.05) compared to CT (Figure 1c).

### 2.2. Polyphenolic Profiles

**Figure 2:**
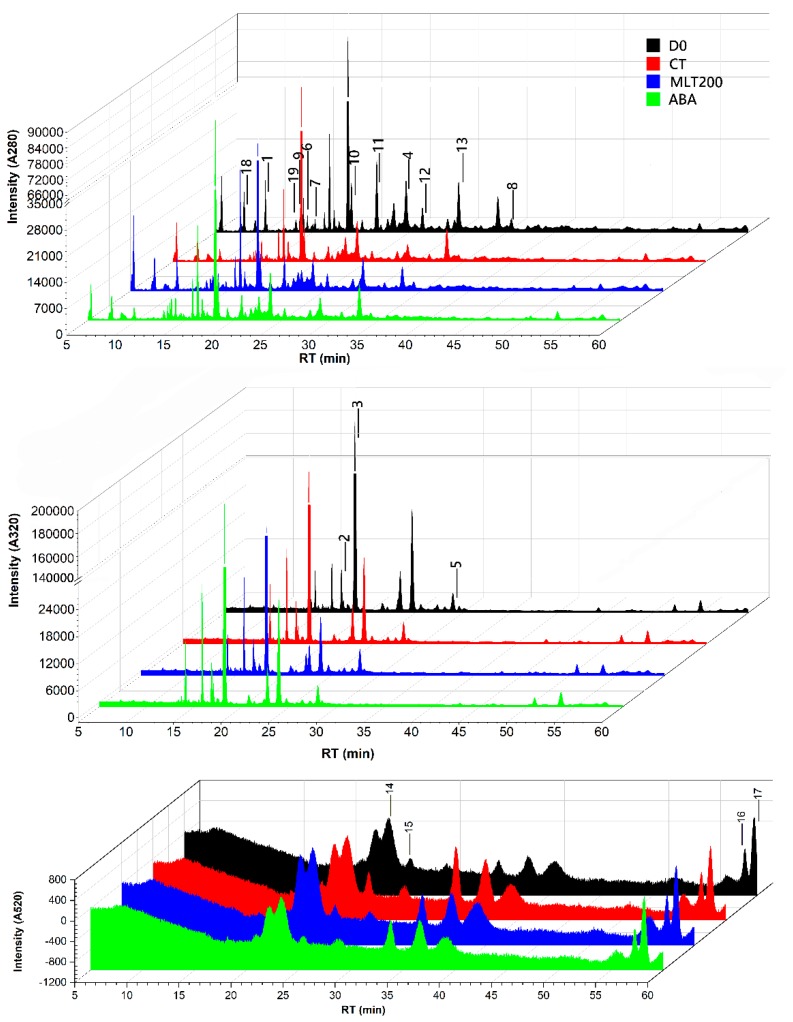
Identification of individual polyphenols in the berry of *Vitis vinifera* cv. ‘Kyoho’. HPLC chromatograms recorded at 280, 320, and 520 nm of the grape extract. The numbered identified peaks are listed in Table 1. To avoid confusion caused by overlapping pictures, only data of D0 (at harvest), CT (control), MLT200 (200 µM melatonin treatment), and ABA (abscisic acid) groups with better effects on polyphenolic biosynthesis promotion were presented in this figure.

**Figure 3:**
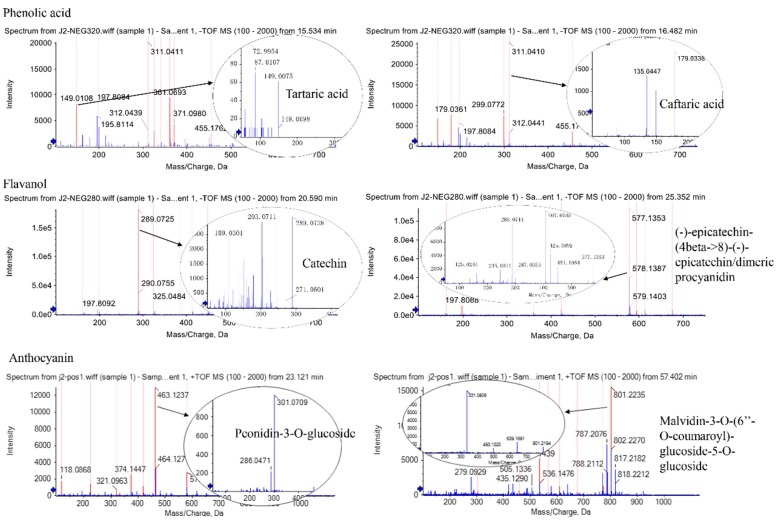
Mass spectra of typical components of phenolic acids, flavanols, and anthocyanins in the berry of Vitis vinifera cv. ‘Kyoho’. The circle at the arrowhead is the secondary mass spectrum of the corresponding substance.

To characterize the polyphenol composition and concentration in response to exogenous treatments, the HPLC-Q-TOF-MS method was employed and a total of 18 polyphenol components were detected (Table 1). By comparison to the reported characteristic ion fragments of grape polyphenols, 16 polyphenols were identified, including four phenolic acids, three flavonols, five flavanols, and four anthocyanins. Figure 2 showed the chromatograms of these compounds at UV 280 nm, UV 320 nm, and UV 520 nm. Several mass spectra of typical components of phenolic acid, flavanol, and anthocyanin are shown in Figure 3.

Phenolic acids were found to be the most abundant polyphenolic compound in grapes (Table 1). Out of the four phenolic acids, caftaric acid was found to be the most abundant component, which accounted for more than 85% of total phenolic acid, followed by fertaric, coutaric, and gallic acid. Consistent with the total phenolic acid content, all identified phenolic acid contents were decreased during storage (Table 1-CT). Among all the treatments, MLT 200 and ABA showed better recovery of all phenolic acids compared to the control (CT). More specifically, MLT200 treatment significantly increased the gallic acid and fetaric acid contents compared to CT (*p* < 0.05). Similarly, NDGA treated grapes showed a significantly higher concentration of gallic acid (*p* < 0.05).

All identified flavonols and flavanols showed significantly lower content after the storage (*p* < 0.05) (Table 1-CT). However, all exogenous treatments showed better recovery of all these components as compared to CT. Among MLT treatments at two different concentrations (100 and 200 μM), MLT200 showed significantly better recovery of all flavonol and flavanol components (*p* < 0.05) (Table 1). More specifically, the concentrations of catechin and epicatechin and their derivatives were tremendously increased in MLT treatment as compared to CT. A similar trend was recorded in that of laricitrin under ABA treatment. Interestingly, polyphenols, such as gallic acid, laricitrin, Kaempferol 3-*O*-glucoronide, proanthocyanidin trimer, and (-)-epicatechin-(4beta->8)-(-)-epicatechin/dimeric procyanidin were found to be significantly higher (*p* < 0.05) in the concentration in nordihydroguaiaretic acid (NDGA) treated grapes compared to D0 (Table 1).

Four anthocyanin compounds were determined, primarily consisted of malvidin and peonidin (Table 1). In agreement with the profiles of total anthocyanin content, the lower concentration of exogenous MLT significantly decreased the content of individual anthocyanins (*p* < 0.05). Furthermore, the peonidin-3-*O*-glucoside compound, which accounted for more than half of the total anthocyanins in the ‘Kyoho’ berry, was found to significantly increase in MLT200 treatment as compared to CT (*p* < 0.05).

### 2.3. Total Phenolic Acid, Flavonol, Flavanol, and Anthocyanin Contents

Polyphenolic content, reflecting the antioxidant capacity of grape berry, is critical to its quality evaluation, particularly to juices and wines. Our results revealed that total phenolic acid content was declined to 562.70 mg kg^−1^ in control after three day (d) storage. Almost all exogenous treatments except ABA was found to significantly decrease the total phenolic acid in grapes (*p* < 0.05) (Table 1). Moreover, since total polyphenol content was mainly determined by phenolic acid, its trend was basically consistent with that of phenolic acid (Table 1). Table 1 showed that the total flavanol wshalf-lost after the storage; however, in the exogenous treated grapes, the contents were significantly recovered (*p < 0.05*) and highest were recorded in NDGA (35.61 ± 1.18 mg kg^−1^) followed by MLT200 (32.80 ± 1.25 mg kg^−1^) treated grapes. The total content of flavonol varied similar to that of flavanol. Anthocyanin content is mainly responsible for the color of the fruit and played a major role in wine formulation. Total anthocyanin content was found to be increased in the control group as compared to D0, which was maintained in ABA treated grapes (Table 1). It was interesting to report that MLT200 showed significantly (*p* < 0.05) higher concentration of total anthocyanin content (10.05 ± 0.25 mg kg^−1^) compared to control and all other treatments.

### 2.4. Expression of Genes Involved in Polyphenolic Biosynthesis

**Figure 4:**
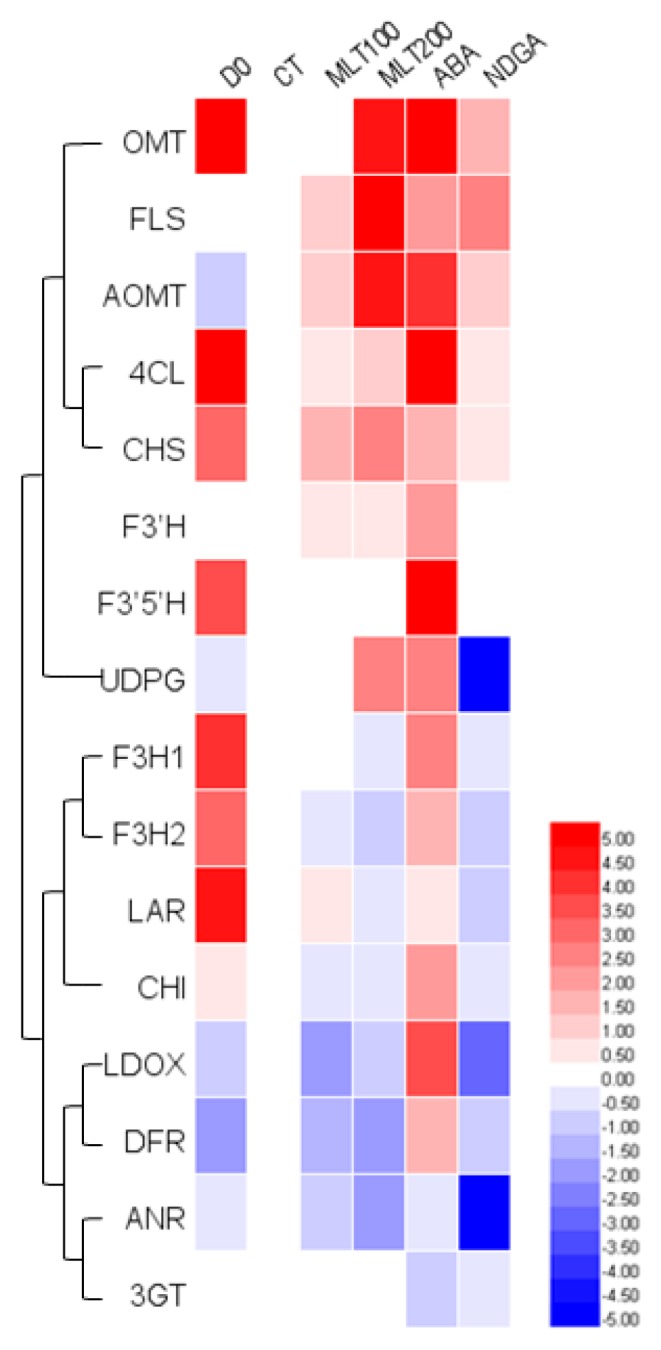
Heatmap of gene expression profiles involved in polyphenolic biosynthesis in the berry of *Vitis vinifera* cv. ‘Kyoho’. The log2 of relative expression levels are shown, measured by RealTime Quantitative Polymerase Chain Reaction (RT-qPCR). CT was set as control.

To better understand how MLT and ABA-induced polyphenol anabolism, RT-qPCR was performed to investigate the gene expression profiles of enzymes involved in polyphenolic biosynthetic pathways (Figure 4). The results indicated that the flavonoid biosynthesis in the ‘Kyoho’ berry was suppressed by the down-regulation of gene expression levels after storage (CT), which was inconsistent with the change in total polyphenol content. Furthermore, almost all gene expression levels were up-regulated by exogenous ABA treatment, except for anthocyanidin 3-*O*-glucosyltransferase (*3GT*) and anthocyanidin reductase (*ANR*); and the upstream flavonoid biosynthetic genes, including 4-coumaroyl-CoA synthase (*4CL*), flavonoid 3′5′-hydroxylase (*F3′5′H*), *LDOX*, anthocyanin 3′-methyltransferase (*OMT*), and flavonoid 3′,5′-methyltransferase (*AOMT*), showed higher transcript levels. On the contrary, most gene expressions in the NDGA group were down-regulated in comparison with ABA. Additionally, most gene expression levels were increased by exogenous MLT, especially *OMT*, flavanol synthase (*FLS*), and *AOMT* genes, which were in agreement with the accumulation of laricitrin and syringetin compounds. And the expression of other genes like *CHI*, flavanone 3β-hydroxylase (*F3H*), *DFR*, *LDOX*, and *ANR* were inhibited by both MLT100 and MLT200 treatments.

## 3. Discussion

Grapes are known to be one of the richest sources of various health-promoting compounds, especially polyphenols [1,21]. *Vitis vinifera* cv. ‘Kyoho’ variety is known for its easy skin peeling effect and considered to possess higher polyphenolic content, thus widely used in wine formulation. In general, our present research results revealed positive changes in polyphenolic content after various postharvested exogenous treatments of grape. 

For fresh-eating, it is essential to check the level of TSS and TA contents before harvesting. TSS and TA are important criteria which determine the maturity and taste. All exogenous treatments in the present study showed different levels of TSS and TA contents. Among all exogenous treatments, MLT maintained the TSS level in postharvested grape, while only a lower concentration of MLT showed significant increase in TA content as compared to control (*p < 0.05*, Figure 1). This might be due to the fact that melatonin inhibited grape respiration, slowed down citric acid circulation, and reduced the degradation of soluble acid, which was confirmed in sweet cherry [22]. The color indicates the ripening stage and berry development. A noticeable change in the berry color was recorded in the control group, while in other exogenous treated groups, it was less predominant. It was previously reported that MLT spraying reduced the under-ripeness and over-ripeness during grape pre-veraison, promoting the berry ripening synchronicity and wines made with it were more fruity, spicy, and sweet [23].

Hilbert et al. [24] identified and detected structures of 18 flavanols from six different *Vitis* varieties using liquid phase mass spectrometry (LC-MS) and liquid chromatography-nuclear magnetic resonance spectroscopy (LC-NMR), respectively. In order to elucidate the effect of these exogenous treatments on polyphenolic profiles of in *Vitis vinifera* cv. ‘Kyoho’ berry during storage, we detected a total of 18 polyphenolic compounds and identified 16 by HPLC-Q-TOF-MS, and determined their relative quantitatively concentrations by HPLC, and investigated the gene expressions involved in the polyphenolic biosynthetic pathway. Moreover, the exogenous melatonin was widely applied in fruit to improve its quality. For instance, grape treated with MLT at pre-veraison stage possessed higher levels of catechins, epicatechins, and peonidin derivatives after maturation [25]. This is consistent with our finding that MLT significantly promoted catechin content (*p* < 0.05). Moreover, the fruit quality and yield of tomato were improved by exogenous MLT, and the contents of phenolics and flavonoids in green-mature tomato were increased by 14.29% and 30.77%, respectively, which were almost twice than that in the red-mature tomato [26]. These results suggested that the polyphenolic biosynthesis was slowed down during fruit senescence, in accordance with the decrease of total polyphenol content in CT after storage (Table 1). In the present study, the polyphenolic contents (phenolic acids, flavonols, flavanols, and anthocyanins) showed differences among different treatments and within polyphenolic contents (Table 1). For example, MLT 200 was found to maintain the total phenolic acid content, while ABA treatment showed an increase in its concentration. However, in the case of total flavonol and flavanol content, NDGA was found to be the best followed by MLT200 and ABA. Anthocyanin, which is mainly responsible for the fruit color, was found to be up-regulated during MLT200 treatment. MLT at lower concentrations was not found much effective in regulating polyphenolic content as compared to a higher concentration. Overall, the total polyphenol content was found to be declined after postharvested storage condition (CT), possibly due to the oxidation process, but maintained and up-regulated by exogenous treatment, especially MLT200 and ABA, respectively (Table 1). This might be attributed to the improved antioxidant capacity in ‘Kyoho’ berry by MLT or ABA, which was further evident in litchi [11] and strawberry [6].

**Figure 5:**
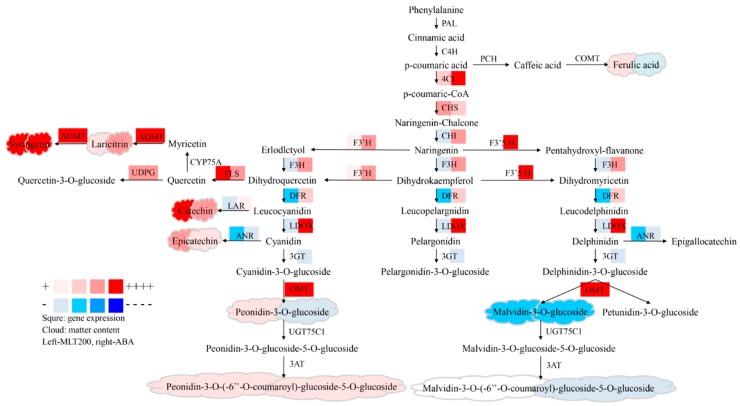
Polyhenolic biosynthesis pathway in berry of *Vitis vinifera* cv. ‘Kyoho’. Red represents up-regulation, while blue represents down-regulation, and white indicates no effect. The deeper the color, the larger the change. And the left reveals MLT200 effect, whereas the right indicates ABA effect. The square and cloud shapes indicate the change in gene expression and component content, respectively.

Additionally, both the polyphenol accumulation and *PAL* expression were observed to be promoted by pre-harvest MLT [12]. In agreement with former studies, our RT-qPCR results showed that *4CL*, *CHS*, *F3′H*, *OMT*, *FLS*, *UDPG* and *AOMT* genes involved in flavonoid biosynthesis were more expressed in different degrees under MLT treatment (Figure 5). In tomato, ABA significantly promoted carotenoid and flavonoid biosynthesis via up-regulating the expression of related genes by 2.08 to 35 times, initiating polyphenol accumulation 2–4 d earlier than control [27]. It was reported that exogenous treatment of ABA in strawberry accelerated flavonoid and anthocyanin biosynthesis, as well as the up-regulated *CHS*, *CHI*, *F3H*, flavonoid 3′-hydroxylase (*F3′H*), and *DFR* expression [20]. And Olivares et al. [28] found that exogenous ABA significantly promoted grape coloration and advanced the harvest time by 37 d. In the present study, total polyphenol content, especially phenolic acid, flavonols, and flavanols were also increased by ABA treatment (Table 1). Particularly, it was revealed that all individual flavonoid compounds except anthocyanins exhibited higher content in ABA and NDGA groups than control (Table 1). Accordingly, *4CL*, *CHS*, *OMT*, *FLS,* and *AOMT* genes involved in polyphenolic biosynthesis showed higher expression in ABA and NDGA groups than control (Figure 5). Expression levels of genes such as *CHI*, *F3H*, *F3′H*, *F3′5′H*, *DFR*, *LDOX*, leucoanthocyanidin reductase (*LAR*), and flavonoid-3-*O*-glucosyltransferase (*UDPG*) were significantly up-regulated in response to exogenous ABA; however significantly and adversely lower in response to NDGA (*p* < 0.05) (Figure 5). A similar outcome was also found in MLT groups, in which not all genes involved in flavonoid biosynthesis had higher expression levels (Figure 4). This paradoxical phenomenon might be due to the profiles of accumulated flavonoids, which were determined from not only the involvement of key genes in the biosynthesis pathway, such as *4CL*, *CHS*, *OMT*, *FLS* and *AOMT*; but also, from the blocking of the ABA biosynthesis pathway by NDGA. It was reported that the polyphenolic biosynthesis and the internal browning in postharvest pineapple were inhibited by the application of 380 µM ABA, due to the improved activity of antioxidant enzymes [29].

**Figure 6:**
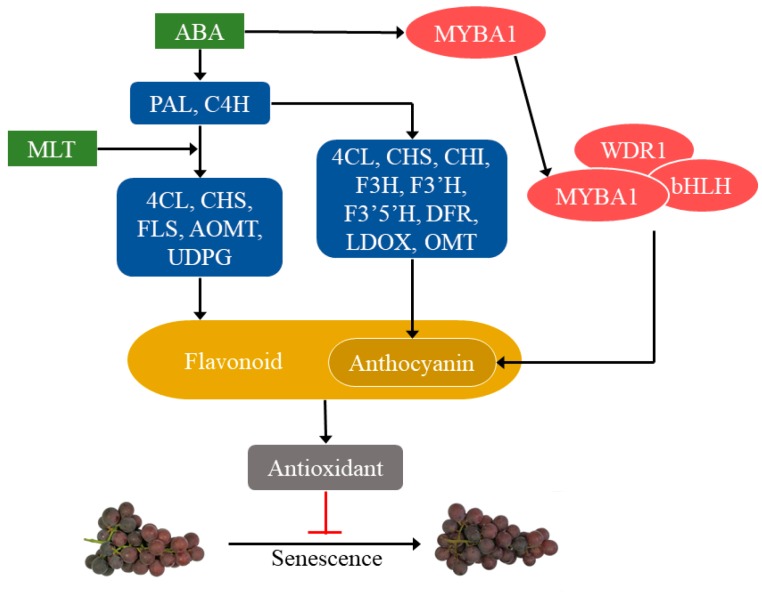
Possible model depicting the mechanism of abscisic acid (ABA) and melatonin (MLT) promoted flavonoid biosynthesis in the berry of *Vitis vinifera* cv. ‘Kyoho’ based on previous and present studies.

It was notable to mention that both MLT and ABA promoted flavonoid biosynthesis; therefore, is there any possible interaction between the influences resulted from exogenous MLT and ABA? Changes of polyphenol contents and gene expressions of vital enzymes involved in polyphenol biosynthesis under ABA and MLT treatments were presented in Figure 5. ABA has been widely recognized as a promotor to polyphenolic biosynthesis, and the increase in the expression of ABA receptor VIPYL1 led to the accumulation of anthocyanin and a series of ABA-responsive gene transcripts in grape berries [30]. The expression of key enzymes in the anthocyanin biosynthesis pathway, including *PAL*, *C4H*, *CHS*, *CHI*, *F3H*, *F3′H*, *DFR*, and *LDOX,* was up-regulated by exogenous ABA in litchi [16] and strawberry [20]. Moreover, the transcription factor MYB could be activated by ABA, then bound to bHLH and WD40 to form a protein complex, which increased anthocyanin biosynthesis [31]. In addition, *FLS* and *UDPG* in the flavonoid biosynthesis pathway were higher expressed by ABA as well [18]. Our results from the present study further confirmed the positive role of ABA in fruit flavonoid biosynthesis (Figure 5). Additionally, the first discovery of MLT receptor in *Arabidopsis thaliana* suggested that MLT was involved in the regulation of physiological attributes, including fruit development and ripening through receptor-mediated signaling cascades [32]. Xu et al. also published that MLT might promote grape ripening by increasing levels of ABA and ethylene [33]. However, very few studies were carried out on the MLT effect on fruit flavonoid biosynthesis so far [12,34]. Our study effectively contributes to narrow down this gap, particularly on grape berries and found that MLT significantly improved flavonoid biosynthesis (*p < 0.05*) via increasing gene expressions of *4CL*, *CHS*, *FLS*, *AOMT,* and *UDPG* (Figure 5). Enhanced flavonoid contents contribute to higher antioxidant activities, thus contributed to the senescence inhibition, quality maintenance of postharvest fruit. Therefore, we postulated a molecular model on the roles of ABA and MLT in polyphenolic biosynthesis in *Vitis vinifera* cv. ‘Kyoho’ berry (Figure 6). Briefly, exogenous ABA promoted the transcription of key enzymes in the main route of polyphenolic biosynthesis pathways, which was then diverged into two roads, leading to higher levels of flavonoids, including anthocyanins. And MLT enhanced flavonoid biosynthesis as well, mainly in a later stage of the pathway. ABA also up-regulated the expression of the MYBA1 (v-myb avian myeloblastosis viral oncogene homolog A1) transcription factor, which further forms a protein complex with transcription factors bHLH and WDR1 to facilitate anthocyanin biosynthesis in grapevine [35]. Increased flavonoid contents contributed to enhanced antioxidant activities and inhibited grape senescence [11]. Our findings provided new insights and made a deeper understanding of flavonoid biosynthesis and gene expression in *Vitis vinifera* cv. ‘Kyoho’ berry under exogenous ABA and MLT. Moreover, since ABA and MLT showed similar effects on promoting the flavonoids biosynthesis, their potential interaction will be further explored in our follow-up study.

## 4. Materials and Methods 

### 4.1. Plant Materials and Experimental Design

**Figure 7:**
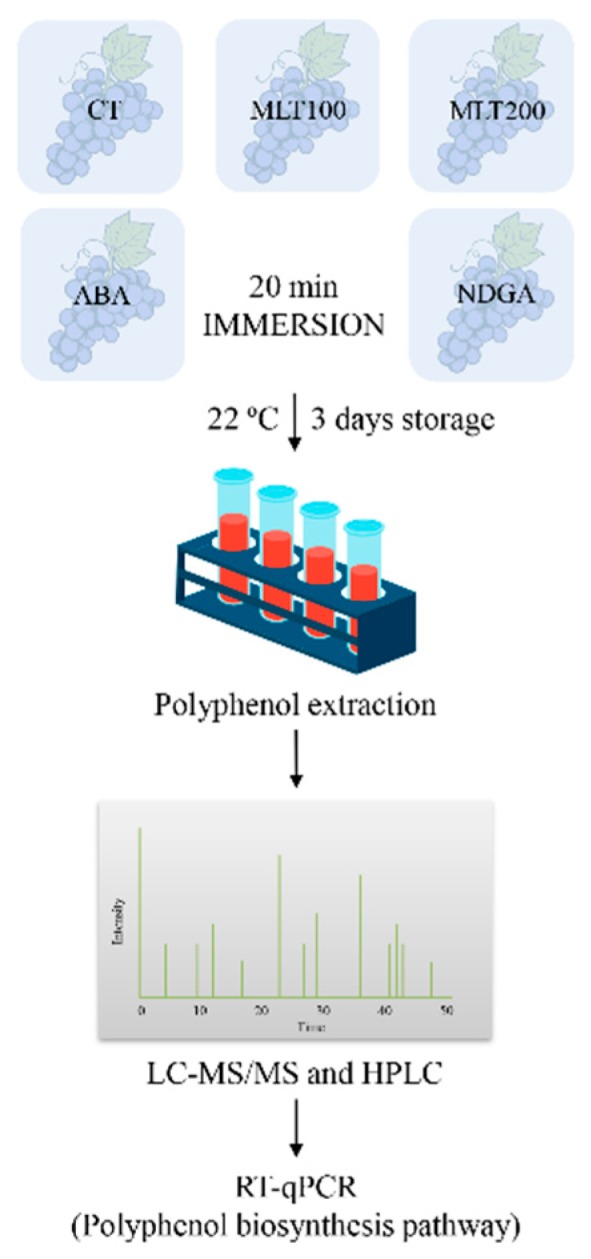
Systematic flow diagram of the experiments.

Three independent biological replications of grape (*Vitis vinifera* cv. ‘Kyoho’) in August with homogenous size were hand-harvested at the same maturity from an organic vineyard in Jinhua city, Zhejiang province, China. For each biological replication, grape bunches were collected in thermocol boxes and delivered to the laboratory in 1.5 h. After acclimatizing at 25 °C for 2 h in the laboratory, the grape bunches were randomly and evenly divided into five groups (three bunches in each group) (Figure 7). Each group was immersed for 20 min in different exogenous chemicals at varied concentrations, i.e., CT (distilled water, control), MLT100 (100 μM melatonin, Macklin Biochemical Company, Ltd., Shanghai, China), MLT200 (200 μM melatonin), ABA (1 mM abscisic acid, Macklin Biochemical Company, Ltd., Shanghai, China) and NDGA (0.5 mM nordihydroguaiaretic acid, Tokyo Chemical Industry, Ltd., Shanghai, China) (Figure 7). Afterward, the grapes were air-dried and stored at room conditions (i.e., 22 ± 3 °C temperature and 60 ± 5% relative humidity) for three d. Berries were randomly removed for determination of total soluble solids (TSS) and titratable acidity (TA), and other berries, including the skin, the flesh, and seeds were ground, mixed, and frozen in liquid nitrogen and stored at −80 °C for further analysis (Figure 7).

### 4.2. Total Soluble Solids (TSS) and Titratable Acidity (TA) Determination

TSS and TA of the fruit juice were measured by a Brix-Acidity Meter (Model PAL-BX/ACID F5, Atago, Japan). Three independent replicates were carried out.

### 4.3. Polyphenols Extraction and Separation

Polyphenols in berry (approximately 2.0 g) were extracted with 1.0 mL of 1% HCl-methanol, at dark 4 °C for 12 h. The resulting supernatant obtained after centrifugation at 10,000× *g* for 15 min was filtered through the 0.22 µm microporous membrane. Polyphenolic compounds were separated by injection into the HPLC system (LC-20AD, SHIMADZU incorporation, Japan) with an AQ-C18 column (water-based, 5 µm, 4.6 × 250 mm, Welch Materials incorporation, Shanghai, China). The elution parameters were as follows: Injection volume, 10 µL; column temperature, 40 °C: Solvent A, 1% phosphoric acid; solvent B, acetonitrile; solvent flow rate, 1.0 mL min^−1^; and the gradient: 0–10 min, 2% B; 10–55 min, 10% B; 55–65 min, 18% B; 65–68 min, 50% B; 68–69 min, 80% B; 69–79 min, 2% B. The signal was monitored at 280, 320 and 520 nm. 

### 4.4. Identification and Quantification of Polyphenols by HPLC and HPLC-Q-TOF-MS

Polyphenols including phenolic acids, flavonoids (including flavones, flavonols, flavanols, flavanones, anthocyanins and so on) were identified using HPLC (as mentioned before) coupled to quadrupole time-of-flight mass spectrometry (Q-TOF-MS) (TripleTOF™ 5600+, SCIEX incorporation, United States) with an electrospray ionization source (ESI) system. Data were generated in negative ion mode with a scan range of 100–1500 m/z and the source voltage at −4.5 kV, the source temperature at 550 °C. The pressure of Gas 1 (Air) and Gas 2 (Air) was 50 psi, whereas that of curtain gas (N_2_) was 35 psi. The molecular formula proposed by PeakView software version 1.2 for different signals were compared with previously reported polyphenolic compounds, especially in *Vitis vinifera*. 

The polyphenolic compounds were quantified by comparison of peak areas with standard calibration curves of the gallic acid (Macklin Biochemical Company, Ltd., Shanghai, China) and epicatechin (Aladdin, Shanghai, China) at 280 and 320 nm, and the cyanidin-3-*O*-glucoside (Macklin Biochemical Company, Ltd., Shanghai, China) at 520 nm. The total concentrations of phenolic acid, flavonol, flavanol, and anthocyanin were obtained by the addition of each identified component concentrations. 

### 4.5. Real-Time Quantitative PCR (RT-qPCR)

Genes involved in the polyphenolic biosynthesis pathway were selected according to Kyoto Encyclopedia of Genes and Genomes (KEGG) pathway database (https://www.genome.jp/kegg/pathway.html). The primers of these genes used in this study were listed in Appendix Table A1. Total RNAs were extracted from powdered grape samples using cetyl trimethyl ammonium bromide (CTAB) protocol described by Gambino et al. [36] and quantified with microplate reader (TECAN, Spark^®^, Männedorf, Switzerland), followed by reverse-transcription to cDNA with a PrimeScript^TM^ RT Reagent Kit (code DRR047 A, TaKaRa, Japan). RT-qPCR was conducted on TB Green™ Premix Ex Taq™ (RR420A, TaKaRa, Japan) according to the instructions mentioned in the product. The glyceraldehyde-3-phosphate dehydrogenase (GAPDH) gene was used as a calibrator to calculate the relative transcriptional levels of genes by the 2^−∆∆CT^ method. Three independent replicates were carried out. And the results were presented as a heat map using the Heml 1.0 software [37]. 

### 4.6. Statistical Analysis 

Experiments were completely randomly designed. All data analysis was performed using SPSS V20.0 (IBM Corp, Armonk, NY, USA) software and presented as mean ± standard deviation with analysis of variance (ANOVA) and Tukey test at a significant level of *p* < 0.05. 

## Figures and Tables

**Table 1:** Identified polyphenolic compounds and their comparative concentrations (mg kg^−1^) in the berry of *Vitis vinifera* cv. ‘Kyoho’.

Peak No.	Compound	Spectrum (nm)	RT (min)	Mw (Da)	Productions	D0	CT	MLT100	MLT200	ABA	NDGA
**Phenolic acids**											
1	Gallic acid	280	9.80	170	125	0.85 ± 0.02 ^c^	0.45 ± 0.09 ^e^	0.65 ± 0.03 ^d^	0.94 ± 0.03 ^b^	0.68 ± 0.03 ^d^	1.22 ± 0.00 ^a^
2	Caftaric acid	320	18.15	312	179,149,135	611.56 ± 4.70 ^a^	538.68 ± 17.25 ^b^	488.60 ± 33.41 ^bc^	511.59 ± 8.23 ^c^	602.93 ± 40.67 ^a^	402.73 ± 2.43 ^d^
3	Coutaric acid	280	23.86	296	163	3.56 ± 0.04 ^a^	2.83 ± 0.12 ^c^	2.29 ± 0.07 ^d^	2.96 ± 0.04 ^c^	3.06 ± 0.03 ^b^	2.08 ± 0.01 ^e^
4	Fertaric acid	320	27.99	326	193,134	21.28 ± 0.16 ^b^	20.74 ± 0.43 ^c^	17.71 ± 0.20 ^e^	24.98 ± 0.15 ^a^	19.67 ± 0.23 ^d^	20.50 ± 0.16 ^c^
Total phenolic acid content						637.25 ± 4.92 ^a^	562.70 ± 17.89 ^b^	509.25 ± 33.71 ^c^	540.47 ± 8.45 ^bc^	626.34 ± 40.96 ^a^	426.53 ± 2.60 ^d^
**Flavonols**											
5	Laricitrin	280	13.65	332	169,125	3.08 ± 0.07 ^c^	1.96 ± 0.32 ^e^	3.46 ± 0.22 ^b^	2.71 ± 0.01 ^d^	3.58 ± 0.54 ^bc^	4.41 ± 0.16 ^a^
6	Syringetin	280	14.88	346	183	1.50 ± 0.00 ^a^	-	-	1.16 ± 0.00 ^b^	0.73 ± 0.00 ^d^	0.88 ± 0.00 ^c^
7	Kaempferol 3-*O*-glucoronide	280	34.27	462	415,311,149	1.94 ± 0.22 ^a^	1.38 ± 0.04 ^b^	1.34 ± 0.10 ^b^	1.97 ± 0.12 ^a^	1.50 ± 0.12 ^b^	2.16 ± 0.10 ^a^
Total flavonol content						6.52 ± 0.29 ^b^	3.34 ± 1.73 ^e^	4.80 ± 0.32 ^d^	5.84 ± 0.13 ^c^	5.81 ± 0.66 ^bc^	7.45 ± 0.26 ^a^
**Flavanols**											
8	Proanthocyanidin trimer	280	13.26	866	866,865,695,575,451,407,287,243,125	2.43 ± 0.05 ^b^	1.34 ± 0.01 ^f^	1.38 ± 0.03 ^e^	2.01 ± 0.01 ^c^	1.45 ± 0.01 ^d^	2.96 ± 0.01 ^a^
9	(-)-epicatechin-(4beta->8)-(-)-epicatechin/dimeric procyanidin	280	18.42	578	425,407,289,245	10.94 ± 0.77 ^a^	6.05 ± 0.58 ^d^	5.42 ± 0.36 ^d^	8.25 ± 0.51 ^b^	8.32 ± 0.00 ^b^	7.23 ± 0.16 ^c^
10	Catechin	280	20.91	290	245	13.57 ± 0.29 ^a^	4.20 ± 0.02 ^f^	5.60 ± 0.30 ^e^	8.24 ± 0.15 ^c^	7.13 ± 0.28 ^d^	8.64 ± 0.18 ^b^
11	(-)-epicatechin-(4beta->8)-(-)-epicatechin/dimeric procyanidin	280	25.34	578	425,407,289,125	4.36 ± 0.09 ^b^	2.37 ± 0.18 ^e^	2.89 ± 0.29 ^d^	4.06 ± 0.09 ^c^	2.78 ± 0.12 ^d^	5.16 ± 0.40 ^a^
12	Epicatechin	280	29.02	290	245,203,109	13.36 ± 0.62 ^a^	6.67 ± 0.21 ^e^	8.15 ± 0.48 ^d^	10.24 ± 0.49 ^c^	8.18 ± 0.26 ^d^	11.62 ± 0.43 ^b^
Total flavanol content						44.66 ± 1.82 ^a^	20.63 ± 1.00 ^f^	23.44 ± 1.46 ^e^	32.80 ± 1.25 ^c^	28.36 ± 0.67 ^d^	35.61 ± 1.18 ^b^
**Anthocyanins**											
13	Peonidin 3-*O*-glucoside	520	23.35	463	301	4.27 ± 0.06 ^d^	5.52 ± 0.10 ^b^	3.19 ± 0.25 ^f^	6.10 ± 0.07 ^a^	4.57 ± 0.09 ^c^	3.62 ± 0.03 ^e^
14	Malvidin 3-*O*-glucoside	520	25.50	493	331	0.32 ± 0.18 ^c^	0.83 ± 0.11 ^a^	0.48 ± 0.05 ^bc^	0.57 ± 0.05 ^b^	0.57 ± 0.04 ^b^	0.45 ± 0.04 ^c^
15	Malvidin 3-*O*-(6″-*O*-coumaroyl)-glucoside-5-*O*-glucoside	520	57.32	801	639,493,331	0.99 ± 0.01 ^b^	1.00 ± 0.04 ^ab^	0.55 ± 0.02 ^d^	1.03 ± 0.02 ^a^	0.84 ± 0.00 ^c^	1.03 ± 0.06 ^ab^
16	Peonidin 3-*O*-(6″-*O*-coumaroyl)-glucoside-5-*O*-glucoside	520	58.19	771	609,463,301	2.67 ± 0.06 ^a^	2.13 ± 0.00 ^c^	1.20 ± 0.05 ^e^	2.35 ± 0.11 ^b^	2.22 ± 0.04 ^b^	2.01 ± 0.02 ^d^
Total anthocyanin content						8.25 ± 0.31 ^c^	9.48 ± 0.25 ^b^	5.42 ± 0.37 ^e^	10.05 ± 0.25 ^a^	8.20 ± 0.17 ^c^	7.11 ± 0.15 ^d^
**Unidentified**											
17	Unknown	280	7.49	370	207	6.50 ± 0.01 ^a^	4.49 ± 0.19 ^d^	4.58 ± 0.09 ^d^	6.19 ± 0.12 ^b^	4.82 ± 0.08 ^c^	6.44 ± 0.04 ^a^
18	Unknown	280	12.86	452	323,89	2.52 ± 0.21 ^a^	1.36 ± 0.05 ^d^	1.28 ± 0.09 ^d^	2.02 ± 0.07 ^b^	1.79 ± 0.01 ^c^	1.69 ± 0.18 ^c^
Total polyphenol content						705.70 ± 7.56 ^a^	602.00 ± 21.11 ^b^	548.77 ± 36.04 ^c^	597.37 ± 10.27 ^b^	675.32 ± 42.55 ^a^	484.83 ± 4.41 ^d^

Data are shown in mean ± standard deviation (n = 3). D0, at harvest; CT, control; MLT100, 100 µM melatonin treatment; MLT200, 200 µM melatonin treatment; ABA abscisic acid treatment; NDGA, nordihydroguaiaretic acid treatment. Different letters (a–e in the table) of the same component under different treatments represent statistically significant differences (*p* < 0.05).

## Abbreviations

*PAL*	phenylalanine ammonia-lyase
*C4H*	cinnamate-4-hydroxylase
*PCH*	p-coumarate 3-hydroxylase
*COMT*	caffeic acid 3-*O*-methyltransferase
*4CL*	4-coumaroyl-CoA synthase
*CHS*	chalcone synthase
*CHI*	chalcone isomerase
*F3′H*	flavonoid 3′-hydroxylase
*F3′5′H*	flavonoid 3′5′-hydroxylase
*F3H*	flavanone 3β-hydroxylase
*DFR*	dihydroflavonol 4-reductase
*LDOX*	leucoanthocyanidin dioxygenase
*3GT*	anthocyanidin 3-*O*-glucosyltransferase
*OMT*	anthocyanin 3′-methyltransferase
*UGT75C1*	anthocyanin 5-*O*-glucosyltransferase
*3AT*	anthocyanidin 3-*O*-glucoside 6″-O-acyltransferase
*CYP75A*	flavonoid 3′,5′-hydroxylase
*AOMT*	flavonoid 3′,5′-methyltransferase
*FLS*	flavonol synthase
*UDPG*	flavonoid-3-*O*-glucosyltransferase
*LAR*	leucoanthocyanidin reductase
*ANR*	anthocyanidin reductase

## Appendix A

**Table A1:** Primers used in RT qPCR.

Genes	Forward Primers (5′-3′)	Reverse Primers (5′-3′)
*4CL*	ACCACCTCCCTCTCCACAC	GCTCCGAGAAAGGAGAACG
*CHS*	ACCCACCTGGATTCTCTCG	GAAGGCTTCCACCAAGCTC
*CHI*	TTGTGTTGGTTCCTCTTGTTC	GCAGACGAATCTCATTCAGTATAG
*F3H1*	CCAATCATAGCAGACTGTCC	TCAGAGGATACACGGTTGCC
*F3H2*	CTGTGGTGAACTCCGACTGC	CAAATGTTATGGGCTCCTCC
*F3′H*	GAGATCAACGGCTACCACATC	CCTGAATTCTAGTGGCTTCTCC
*F3′5′H*	GCTGGCACTAGAATGGGAATAG	CTCAACTCCATCCGGCATTT
*DFR*	TTGTAATGGTCAATGTGCC	CATGCAGAGACCACCTTG
*LDOX*	ACCTTCATCCTCCACAACAT	AGTAGAGCCTCCTGGGTCTT
*3GT*	TCATGAAGACCAGACCCTTA	TGTTGCTACTACGGGGTCTA
*OMT*	AGATAAAGAAGCGTACCAAA	GCTCATGGTAGTTGAGGTAG
*LAR*	CGGTTGGTAGCATTAAGAGGTTC	GGAGTTGCAGCAGATGTAGGTGT
*ANR*	TTGATGGGACAGGTCTGGTTG	AGTGTCTTGGAGGCAGGATAGC
*FLS*	CCAGCCAATCCTTCAAACTT	TTTGCCATTTGCATTGTGTA
*UDPG*	TGCTACCTAAGGCGACTG	GCTTGGATTTGAGATCATTGG
*AOMT*	AGGTGTGGAGCATAAGATCA	TTATCGTAAGCGATTATGCC
